# Multiparametric Analysis of Nasal Inspiratory Pressure (SNIP) in Duchenne Muscular Dystrophy: A Case‐Control Study With Healthy Subjects

**DOI:** 10.1002/ppul.71344

**Published:** 2025-10-23

**Authors:** Ilsa P. Santos, Layana Marques, Jessica D. M. da Fonseca, Mario E. Dourado, Matías Otto‐Yáñez, Rodrigo Torres‐Castro, Francesca Pennati, Andrea Aliverti, Guilherme A. F. Fregonezi, Vanessa R. Resqueti

**Affiliations:** ^1^ Laboratório de Inovação Tecnológica em Reabilitação, Departamento de Fisioterapia Universidade Federal do Rio Grande do Norte (UFRN) Natal Rio Grande do Norte Brazil; ^2^ Departamento de Fisioterapia, Universidade Federal do Rio Grande do Norte (UFRN) PneumoCardioVascular Lab/HUOL, Hospital Universitário Onofre Lopes Natal Rio Grande do Norte Brazil; ^3^ Departamento de Medicina Integrada, Hospital Universitário Onofre Lopes Universidade Federal do Rio Grande do Norte (UFRN) Natal Rio Grande do Norte Brazil; ^4^ Grupo de Investigación en Salud, Funcionalidad y Actividad Física (GISFAF), Kinesiología, Facultad de Ciencias de la Salud, Universidad Autónoma de Chile Santiago Chile; ^5^ Department of Physical Therapy, Faculty of Medicine University of Chile Santiago Chile; ^6^ Dipartimento di Elettronica Informazione e Bioingegneria, Politecnico di Milano Milan Italy

**Keywords:** maximal respiratory pressures, muscle weakness, neuromuscular diseases, respiratory function tests, respiratory muscles

## Abstract

**Background:**

Individuals with Duchenne Muscular Dystrophy (DMD) exhibit respiratory muscle changes leading to fatigue and weakness, and assessing relaxation rates and contractile properties may help detect early fatigue.

**Aim:**

To non‐invasively assess inspiratory muscle relaxation and contractile rates using sniff nasal inspiratory pressure (SNIP) parameters in DMD subjects and compare them with matched healthy controls.

**Methods:**

A case‐control study of 32 DMD male subjects and 32 age‐matched healthy controls (12.7 ± 5.1 years). All subjects underwent spirometry, maximal respiratory pressures, and SNIP test. We calculated the maximum relaxation rate (MRR), decay constant (τ), and maximum rate of pressure development (MRPD) from the SNIP curve.

**Results:**

The DMD group had significantly lower MRR (5.9 [5.1–6.9] vs. 8 [6.9–10.3] %/ms, *p* = 0.001), lower MRPD (−0.38 [−0.47 to −0.26] vs. −0.62 [−0.52 to −0.80] cmH_2_O/ms−1, *p* = 0.001), and higher τ (65.7 [50.7–78.1] vs. 40.5 [30.2–48.7] ms, *p* = 0.001). ROC curves showed that SNIP parameters effectively distinguish DMD from healthy subjects (SNIP [AUC 0.94, *p* < 0.001], MRR [AUC 0.86, *p* < 0.001], τ [AUC 0.92, *p* < 0.001], and MRPD [AUC 0.89, *p* < 0.001]).

**Conclusions:**

DMD subjects show impaired inspiratory muscle contraction and relaxation, indicating early muscle weakness or fatigue. SNIP‐derived parameters may help in the early identification of inspiratory muscle dysfunction in DMD, potentially contributing to clinical detection and intervention.

## 1 Introduction

Duchenne Muscular Dystrophy (DMD) is an X‐linked inherited genetic disease due to a mutation in the gene responsible for encoding the dystrophin protein [1]. The global overall mean prevalence of the disease was 19.8 cases per 100,000 male live births [2]. The clinical manifestations of DMD begin in early childhood with progressive muscle weakness as the main symptom, which leads to severe complications, including disability and premature death [3, 4]. Muscle weakness progresses until the use of a wheelchair becomes necessary before adolescence, while simultaneously or subsequently respiratory, orthopedic, and cardiac complications arise [5]. Respiratory failure and/or pulmonary infection are common causes of death [6, 7].

Therefore, the constant assessment of respiratory muscle strength helps to guide therapy recommendations and patient management. Measurements of maximum static inspiratory (MIP) or expiratory (MEP) pressures at the mouth allow for a simple assessment of global respiratory muscle strength in a clinical setting. As respiratory muscle weakness progresses, muscle strength loss occurs before the reduction in lung volume is detected [8, 9]. Considering that maximum respiratory pressure measurements are volume‐dependent, alternative measures of muscle strength should be adopted. An alternative and/or complementary test to evaluate inspiratory force is the sniff nasal inspiratory pressure (SNIP) test, which records nasal inspiratory pressure during sniffing [10]. This test has the advantage of measuring inspiratory pressure during a physiological maneuver, not volume‐dependent, that is, easily performed, even by children with neuromuscular diseases, and may prove to be more reliable and sensitive in this population [11, 12]. Additionally, the relaxation rates and contractile properties of the inspiratory muscles derived from the SNIP curve can help identify early respiratory muscle fatigue.

The relaxation rates obtained from a maximum inspiration can be described by the Maximum Relaxation Rate (MRR) [13]; and by the time constant of the pressure decay curve (τ, tau) after maximum voluntary contraction. Esau et al. demonstrated that these relaxation parameters are significantly altered by the development of inspiratory muscle fatigue and have been used as predictive indices of respiratory muscle fatigue by several authors [14, 15, 16].

In addition, the contractile properties of inspiratory muscles can be described by the Maximum Rate of Pressure Development (MRPD), which can be altered in cases of fatigue and has been used as an index to assess respiratory muscle function [16].

We hypothesize that parameters related to the relaxation rates and contractile properties of inspiratory muscles are altered in individuals with DMD, serving as early indicators of inspiratory muscle weakness. Therefore, the present study aimed to analyze these parameters through SNIP test analyses in DMD subjects compared to healthy individuals, and to assess their sensitivity and specificity in differentiating between the two groups.

## 2 Methods

This case‐control study was carried out at the Pneumocardiovascular Laboratory, Hospital Universitário Onofre Lopes (HUOL)/UFRN, Natal, Brazil. The sample was recruited for convenience and divided into two groups: the DMD Group, composed of individuals referred from the Neuromuscular Diseases Clinic of HUOL/UFRN, and the control group, composed of healthy individuals matched by age and gender. This study was reported following the “Strengthening the Reporting of Observational Studies in Epidemiology” (STROBE) statement [17]. The inclusion criteria for the DMD group were a molecular and clinical diagnosis consistent with DMD, age over 7 years, a sufficient level of cognition to understand the procedures to be performed, being free of associated infectious lung diseases, having no history of tracheostomy, noninvasive ventilation < 15 h per day, and not using sedatives. The control group included healthy subjects, with forced vital capacity (FVC) values greater than 80% and a forced respiratory volume in the first second to FVC ratio (FEV_1_/FVC) greater than 85% or 0.7 L liters, with similar age to the DMD individuals, and a sufficient level of cognition to understand the procedures performed. The exclusion criteria for both groups were refusing to participate in any study stage and/or not completing any research procedure.

### 2.1 Ethical Aspects

This project was approved by the Research Ethics Committee of HUOL/UFRN, protocol number 1.817.836. Research participants and their guardians received clarification about the project and guaranteed anonymity of the volunteers, where all signed the Informed Consent Form and the Informed Consent Form with information on the purposes, risks and benefits of research for both individuals and legal caregivers. All participants signed the informed consent following the Declaration of Helsinki.

### 2.2 Study Design

**Figure 1:**
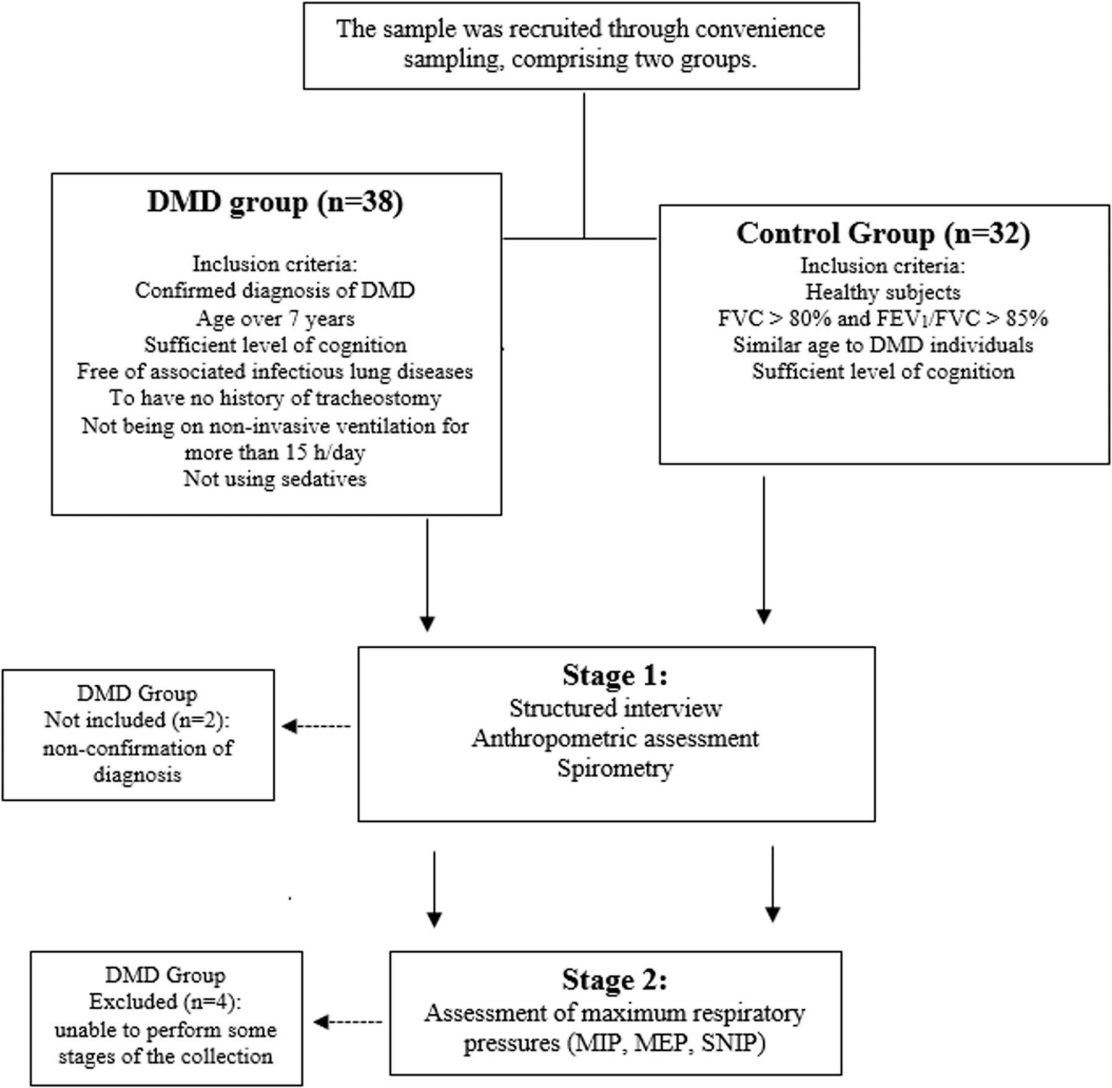
Study flowchart.

The research protocol was initiated after the participants and their legal guardians (for minors) signed the informed consent form. Assessments were conducted in two stages on a single day by a trained assessor. The first stage included a structured interview (sociodemographic data and previous diseases), anthropometry data (weight, height, and body mass index [BMI] and spirometry). In the second stage, maximum respiratory pressures (MIP, MEP, and SNIP) were performed (Figure 1).

### 2.3 Assessment Tools

#### 2.3.1 Pulmonary Function

Spirometry was performed using the Koko nSpire spirometry software (LongMont, USA), according to the technical procedures, as well as the criteria for acceptability, reproducibility, interpretation, and standardization of the equipment, recommended by the American Thoracic Society/European Respiratory Society (ATS/ERS) [18]. The variables evaluated were FVC, final expiratory volume in 1 s (FEV_1_), and the FEV_1_/FVC ratio. The predicted values were calculated using the reference values proposed by Mallozi and published by Pereira [19] for patients aged 7−18 years and Pereira et al. [20], for those over 18 years old.

#### 2.3.2 Respiratory Muscle Strength

Maximum respiratory pressures were assessed using a Digital Manovacuometer V0.2 (NEPEB‐LabCare/UFMG, Belo Horizonte‐MG, Brazil). The measurement of MIP and MEP adhered to the technical criteria for acceptability and reproducibility as per the standards and recommendations of the ERS [9]. For each assessment, three to five maneuvers were performed, with at least three acceptable and two reproducible maneuvers, whose values cannot differ by more than 10%, being considered the highest obtained value. The obtained values were compared to reference values for the Brazilian population using the equation proposed by Lanza et al. [21] for patients aged 7−18 years and Araújo et al. [22] for those over 18 years old.

For the SNIP test, a nasal plug was used to occlude one nostril while the other remained free to allow air passage. The maneuver was performed following the technical criteria recommended by the ATS/ERS [18] guidelines. The subjects executed a maximum and rapid inspiratory effort, similar to a sniff. Ten maneuvers were performed from the functional residual capacity (FRC) with an interval of 30 s between each maneuver. The maneuver with the highest pressure peak was selected, with a total duration of fewer than 500 ms, peak pressure maintained for less than 50 ms, and a sniff pressure waveform with a smooth decay curve [23]. We adopted the reference values proposed by Marcelino et al. [24], for patients aged 7−18 years old and Araújo et al. [25], for those over 18 years old.

#### 2.3.3 Snip Curve Analysis

The SNIP Curve Analysis was analyzed by the MATLAB 2014 software (The MathWorksInc, Natick, MA, USA) to calculate the parameters derived from the SNIP curve. MRPD was expressed as cmH_2_O/ms, and calculated as the negative peak of the first derivative of the pressure‐time curve, and the value normalized by the SNIP peak also was analyzed [13, 26].

The time constant (τ) was also calculated. A graph of the natural logarithm of pressure versus time produces a straight line over the first 50%−70% of the pressure decay curve, indicating a monoexponential decay, with a time constant τ (τ = 1/slope). The correlation coefficient of the individual regression line (ln(pressure) vs. time) had to be ≥ 0.96 for a tau measurement to be accepted [13, 27].

The MRR was calculated as the ratio between the peak of the first derivative of pressure‐time curve (dP/dt) normalized to the sniff pressure peak, and expressed as a percentage of the pressure drop per 10 ms [28].

### 2.4 Statistical Analysis

The sample size was determined considering SNIP as the main variable. The five subjects initially evaluated in each group were analyzed using a hypothetical *t*‐test with mean and standard deviation (SD) for the Duchenne (44.6 ± 9.3 cmH_2_O) and Healthy (102.6 ± 10.8 cmH_2_O) groups, with an effect size of 1.45. A sample of 40 subjects (20 in each group) was estimated using an alpha error of 0.05 with bilateral distribution and a test power of 95%.

The distribution of the data was assessed using the Shapiro−wilk test. Data were expressed as mean and standard deviation for parametric distribution and confidence interval or median and interquartile range 25%−75% for non‐parametric distribution. The comparison was performed using the *T*‐test and the Mann−Whitney *U* test for the inter‐group and intra‐group analysis. Additionally, the ROC curve was calculated to assess the sensitivity/specificity of MRR, τ and MRPD. The data were analyzed using the statistical program GraphPadPrism 6.0 (San Diego, California). A significance level of 5% (*p* < 0.05) was adopted.

## 3 Results

Thirty‐eight male individuals were recruited. Of these, two did not meet the inclusion criteria due to non‐confirmation of DMD diagnosis, and four were excluded as unable to perform some stages of the collection. Therefore, 32 individuals with DMD were evaluated and compared to 32 healthy individuals matched for sex and age (12.7 ± 5.1 years). Compared to healthy individuals, individuals with DMD had lower lung function parameters (*p* < 0.001) and respiratory muscle strength (*p* < 0.001), as shown in Table 1, which presents the characterization of the sample.

**Table 1:** Sample characterization: anthropometric data, pulmonary function, respiratory muscle strength.

	DMD group	Healthy group	*p*
Subjects, n	32	32	—
Age, years	12.7 ± 5.1	12.7 ± 5.1	—
Height, m	1.43 ± 17.3	1.51 ± 19.3	0.09
Weight, kg	39 ± 18.4	46.6 ± 19.5	0.08
BMI, kg/m^2^	19.1 ± 5.2	20.7 ± 4	0.10
FVC, (L)	1.77 ± 0.67	3.05 ± 1.33	0.001
FVC%	72.2 ± 24	100.7 ± 13.8	0.001
FEV1, (L)	1.45 ± 0.72	2.58 ± 1.07	0.001
FEV_1_%	63.5 ± 25.8	91.4 ± 21.6	0.001
FEV_1_/FVC	0.80 ± 0.15	0.84 ± 0.06	0.001
MIP, cmH_2_O	53.4 ± 17.9	105.9 ± 23.9	0.001
MIP, %	53 ± 21.1	105.2 ± 28.9	0.001
MEP, cmH_2_O	50.3 ± 16.1	118.5 ± 27.8	0.001
MEP, %	46.3 ± 15.9	107.8 ± 26.1	0.001
SNIP, cmH_2_O	50.8 ± 18.5	92.4 ± 19.5	0.001

*Note:* Data are presented as mean and standard deviation.

Abbreviations: BMI, body mass index; FVC, forced vital capacity; % predicted, percentage of predicted value; FEV_1_, forced expiratory volume in the first second; MEP, maximal expiratory pressure; MIP, maximal inspiratory pressure.

**Figure 2:**
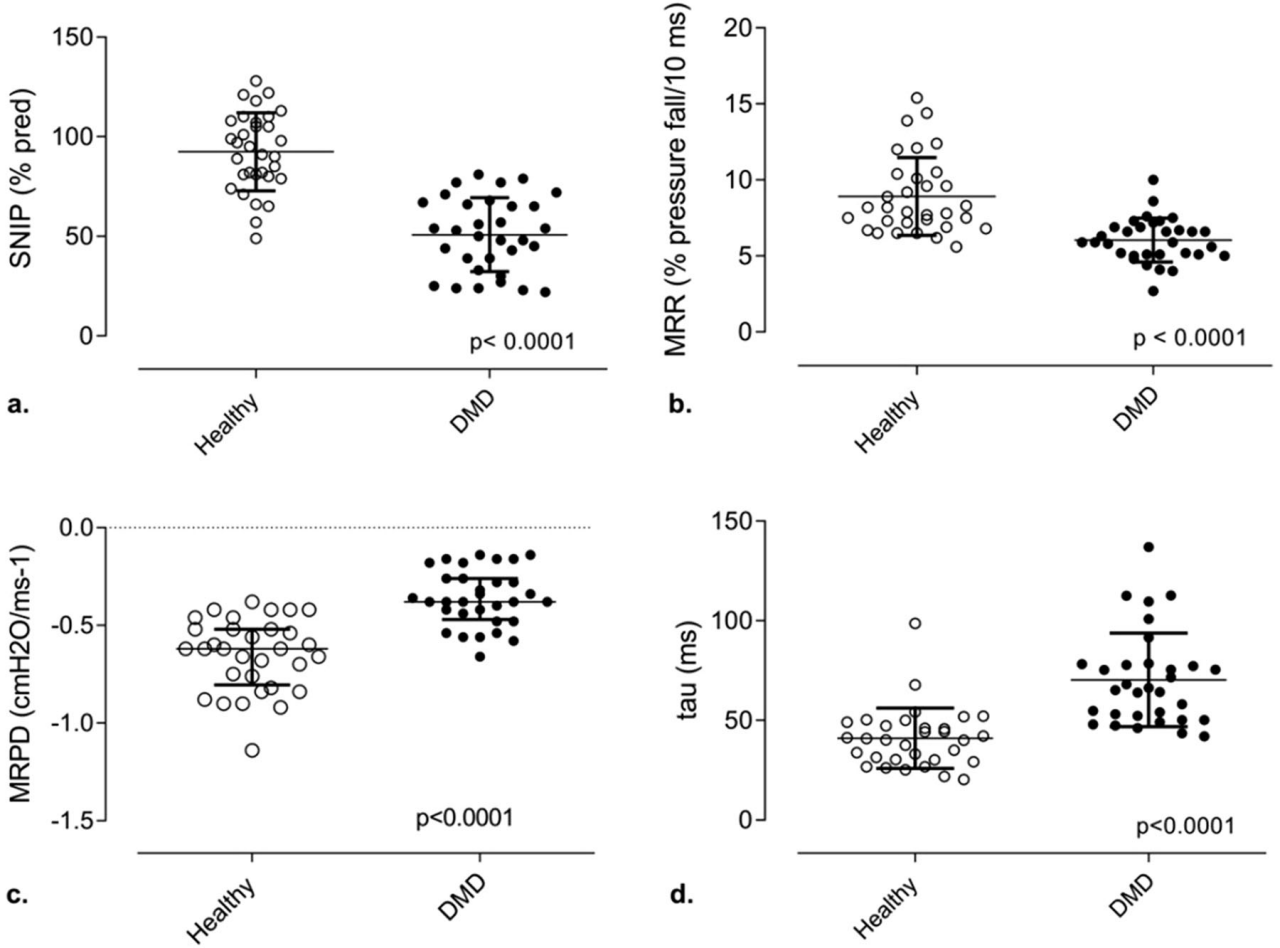
Comparisons between the parameters obtained from the sniff nasal inspiratory pressure (SNIP) curve (a) percentage of predicted SNIP test (SNIP % pred), (b) maximum relaxation rate (MRR), (c) maximum rate of pressure development (MRPD), (d) tau (τ)] between DMD and healthy subjects. cmH_2_O, centimeters of water; ms, milliseconds.

Figure 2 shows the parameters of relaxation rates and contractile properties extracted from the SNIP curve. Subjects with DMD showed an MRR (5.9 [5.1 to 6.9] vs. 8 [6.9 to 10.3] %/ms, *p* = 0.001) and MRPD (−0.38 [−0.47 to −0.26] vs. −0.62 [−0.52 to −0.80] cmH2O/ms, *p* = 0.001) significantly lower in absolute value than healthy individuals, as well as presenting a higher τ value (65.7 [50.7−78.1] vs. 40.5 [30.2−48.7] ms, *p* = 0.001).

In addition, we performed a subanalysis of the 14 individuals with DMD who still had FVC and maximum respiratory pressures within the normal range and independent mobility, comparing their results with a control group of the same age range (Table 2).

**Table 2:** Multiparametric analysis derived from the SNIP curve in individuals with DMD who still have normal maximum respiratory pressures.

	DMD	Healthy	*p*
Subjects, (*n*)	14	14	—
Age, (years)	10 [9–12.1]	10 [9–12.1]	—
FVC, %	90 [80.1–99.9]	108 [95.2 −119.5]	0.03
MIP, %	89 [85–93.8]	124.5 [110.5–138.5]	< 0.001
MEP, %	89.5 [85–92.2)	118 [105.8–133.3]	0.01
SNIP, %	92 [85.1–100.2]	105.5 [92.5–119.5]	0.01
MRR, %/ms	6.0 [5.3–7.2]	8.2 [7–9.9]	< 0.001
MRPD, cmH_2_O/ms	3.8 [3.4–4.8]	6.4 [5.4–8.2]	< 0.001
τ, ms	61.2 [49.3–88]	33.5 [27.4–46.9]	< 0.001

*Note:* Data are shown as median [25–75th percentile].

Abbreviations: τ, tau; % percentage of predicted value; cmH_2_O, centimeters of water; FVC, forced vital capacity; MEP, maximal expiratory pressure; MIP, maximal inspiratory pressure; MRPD, maximum rate of pressure development; MRR, maximum relaxation rate; ms, milliseconds.

**Figure 3:**
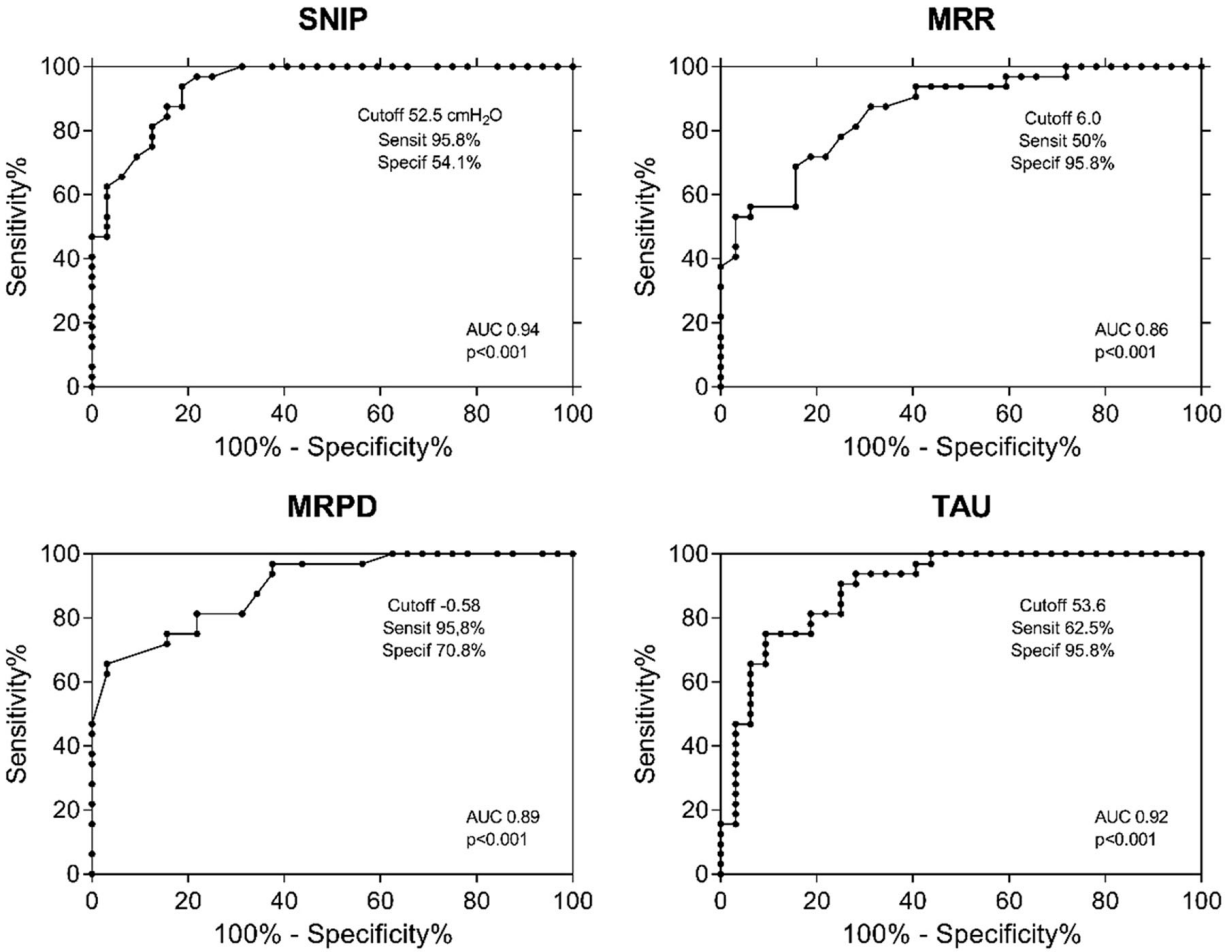
ROC curve: SNIP Peak Pressure, MRR, MRPD, and tau in DMD and healthy individuals. The area under the curve (AUC) above 0.80 associated with a *p* < 0.05 leads to the conclusion that these variables show a good discriminative capacity for identifying muscle fatigue/weakness.

The sensitivity/specificity, cutoff points, and *p* values for each variable, including SNIP, MRR, MRPD, and τ are presented in Figure 3. All these variables demonstrate a strong discriminatory ability between individuals with the disease and healthy controls, as evidenced by the area under curve (AUC).

## 4 Discussion

Our main findings indicate that, compared to healthy subjects, individuals with DMD exhibit: (I) lower lung function and respiratory muscle strength; (II) reduced MRR and MRPD; (III) increased tau; (IV) significant changes in SNIP‐derived parameters even at early disease stages, suggesting their potential role in discriminating individuals with muscle fatigue from those without it.

The MRR has been sparsely studied for about 25 years [28, 29]. This fatigue index is collected from sniff maneuvers, which were originally measured using esophageal or trans‐diaphragmatic pressure, both of which are invasive methods [15]. Fitting and Leuenberger [30] were the first authors to study the relaxation rate in a single case. These authors evaluated the effects of procainamide, an anti‐myotonic drug, on the diaphragm through transdiaphragmatic pressure. Kyroussis et al. [28, 29] and García‐Rio et al. [31] found that the MRR of the respiratory muscles measured using the SNIP, a non‐invasive maneuver, is highly correlated with the measurement made using esophageal pressure and has been reported as a predictor of muscle fatigue. In our study, individuals with DMD had significantly lower MRR values than their healthy counterparts. Although MRR has never been studied in DMD, our results are consistent with studies in other neuromuscular diseases. Evangelista et al. [15] demonstrated a lower MRR in Myotonic Dystrophy type 1 (DM1) patients compared to controls (*p* = 0.03), and Sarmento et al. [16] reported lower MRR (*p* < 0.001) in individuals with Amyotrophic Lateral Sclerosis (ALS) compared to healthy individuals.

Literature on MRPD and τ measurements in patients with neuromuscular diseases is still scarce. It is known that loss of muscle strength or fatigue decreases the speed of muscle contraction, leading to an increase in contraction time and relaxation time. Our results show that τ is significantly increased and MRPD significantly decreased in individuals with DMD compared to healthy individuals. Sarmento et al. [16] reported similar findings, with lower MRPD (*p* < 0.001) and higher τ (*p* < 0.001) in individuals with ALS compared with healthy controls.

Furthermore, considering that individuals with DMD experience progressive loss of respiratory muscle strength from childhood onwards, our study showed changes in SNIP‐derived parameters compared to healthy individuals, even in the early stages of disease progression, when other pulmonary parameters are still within the normal range and individuals are not yet confined to a wheelchair.

Marcelino et al established reference values for nasal inspiratory pressure in healthy Brazilian children. The lower limit of normal for this group was approximately 56−57 cmH₂O in the children in this study [24]. Therefore, our study provided a subanalysis of 14 individuals with DMD who, although they have lower MIP, MEP, SNIP, and FVC values than healthy individuals, are still within the normal range. These subjects already show changes in SNIP‐derived variables, even when inspiratory muscle strength is still above the lower normal limit, which may suggest the presence of respiratory muscle fatigue at an early stage.

The results of the ROC curves show that all parameters derived from the SNIP curve effectively discriminate DMD from healthy individuals. Peak pressure and MRPD showed greater sensitivity, making them more effective in identifying individuals with muscle fatigue. In contrast, τ showed greater specificity, indicating its strong discriminatory power in identifying individuals without muscle fatigue. In addition, the ROC curves provided valuable information on the cut‐off points for each variable.

The loss of muscle strength during fatigue leads to a reduced rate of muscle contraction and an extended recovery period as adaptive mechanisms. These changes are related to intracellular and metabolic factors, such as a decline in the calcium uptake by the sarcoplasmic reticulum, ATP depletion and intracellular acidosis [15, 16, 32, 33, 34]. Therefore, the observed decrease in MRR, MRPD, and the increase in τ seem to be adaptive mechanisms and may serve as early indicators of fatigue onset, as these changes precede the reduction in maximum inspiratory pressure. To our knowledge, no previous study has evaluated parameters derived from the SNIP in individuals with DMD. Therefore, our results are pioneering in identifying contraction and relaxation rates from SNIP measurement for early detection in this population, potentially contributing to a better understanding of inspiratory muscle fatigue in individuals with DMD.

As a limitation, we point out the scarcity of studies in the scientific literature that evaluate the relaxation and contraction rates of inspiratory muscles in both healthy individuals and individuals with diseases, which complicates the comparison of our results. Additionally, we highlight the potential need for specialized software, which may not be available in all healthcare settings. Therefore, additional studies using the SNIP test to evaluate the relaxation rates and contractile properties of the respiratory muscles, as well as the use of electromyography in these muscles, are essential to provide further clarification on the subject. Furthermore, studies involving larger and more diverse DMD populations, including longitudinal follow‐up and evaluation of therapeutic needs, are necessary to validate the use of SNIP parameters in clinical practice.

In terms of clinical applicability, the calculation of SNIP curve parameters is simple and provides valuable information about the state of the respiratory muscles. This approach may allow for the early detection of fatigue before respiratory failure occurs, as well as the early implementation of new therapeutic interventions before the onset of pressure drop in the SNIP curve [16].

## 5 Conclusion

Individuals with DMD show changes in relaxation rates and contractile properties of inspiratory muscles, as derived from the SNIP curve. These changes are detectable even in the early stages of disease progression when compared to healthy individuals. In addition, these parameters demonstrate high sensitivity (MRPD and peak SNIP) and specificity (τ) for identifying individuals with muscle fatigue from those without. These findings suggest that relaxation rates and contractile properties of inspiratory muscles may serve as valuable markers to complement conventional assessment of patients with DMD, from the onset of their ability to perform SNIP tests, potentially contributing to clinical detection and intervention.

## Author Contributions

**Ilsa P. Santos:** conceptualization, methodology, formal analysis, investigation, data curation, writing − original draft, writing−review and editing, visualization, project administration. **Layana Marques:** methodology, investigation, data curation. **Jessica D. M. da Fonseca:** writing − review and editing; **Mario E. Dourado:** resources; investigation. **Matías Otto‐Yáñez:** writing − review and editing. **Rodrigo Torres‐Castro:** writing − review and editing. **Francesca Pennati:** software, writing−review and editing. **Andrea Aliverti:** software; writing − review and editing. **Guilherme A. F. Fregonezi:** resources, writing − review and editing, funding acquisition. **Vanessa R. Resqueti:** conceptualization, methodology, formal analysis, resources, writing − review and editing, supervision, funding acquisition.

## Conflicts of Interest

The authors declare no conflicts of interest.

## Data Availability

The data that support the findings of this study are available from the corresponding author upon reasonable request.
